# Rapid Identification and Investigation of an HIV Risk Network Among People Who Inject Drugs –Miami, FL, 2018

**DOI:** 10.1007/s10461-019-02680-9

**Published:** 2019-09-25

**Authors:** Hansel Tookes, Tyler S. Bartholomew, Shana Geary, James Matthias, Karalee Poschman, Carina Blackmore, Celeste Philip, Edward Suarez, David W. Forrest, Allan E. Rodriguez, Michael A. Kolber, Felicia Knaul, Leah Colucci, Emma Spencer

**Affiliations:** 1 University of Miami, 1120 NW 14th St #860, Miami, FL 33136 USA; 2 Florida Department of Health, Division of Disease Control and Health Protection, Bureau of Communicable Diseases, HIV/AIDS Section, Tallahassee, FL USA; 3 Centers for Disease Control and Prevention, National Center for HIV/AIDS, Viral Hepatitis, STD and TB Prevention, Division of STD Prevention, Atlanta, GA USA; 4 Centers for Disease Control and Prevention, National Center for HIV/AIDS, Viral Hepatitis, STD and TB Prevention, Division of HIV/AIDS Prevention, Atlanta, GA USA

**Keywords:** People who inject drugs, HIV, Molecular surveillance, Outbreak investigation

## Abstract

Prevention of HIV outbreaks among people who inject drugs remains a challenge to ending the HIV epidemic in the United States. The first legal syringe services program (SSP) in Florida implemented routine screening in 2018 leading to the identification of ten anonymous HIV seroconversions. The SSP collaborated with the Department of Health to conduct an epidemiologic investigation. All seven acute HIV seroconversions were linked to care (86% within 30 days) and achieved viral suppression (mean 70 days). Six of the seven individuals are epidemiologically and/or socially linked to at least two other seroconversions. Analysis of the HIV genotypes revealed that two individuals are connected molecularly at 0.5% genetic distance. We identified a risk network with complex transmission dynamics that could not be explained by epidemiological methods or molecular analyses alone. Providing wrap-around services through the SSP, including routine screening, intensive linkage and patient navigation, could be an effective model for achieving viral suppression for people who inject drugs.

## Resumen

La prevención de brotes de VIH entre las personas que se inyectan drogas sigue siendo un desafío para poner fin a la epidemia de VIH en los Estados Unidos. El primer programa legal de servicios de intercambio de jeringas (SSP como se conoce con sus siglas en inglés) en Florida, implementó pruebas rutinarias en 2018 que condujo a la identificación de diez seroconversiones anónimas de VIH. El SSP colaboró ​​con el Departamento de Salud para realizar una investigación epidemiológica. Siete seroconversiones agudas de VIH fueron identificadas y se vincularon a la atención médica (86% en 30 días) y lograron la supresión viral (media 70 días). Seis de los siete individuos están vinculados epidemiológicamente y/o socialmente con al menos otras dos seroconversiones. El análisis de los genotipos del VIH reveló que dos individuos están conectados molecularmente a una distancia genética de 0.5%. Identificamos una red de riesgo con una dinámica de transmisión compleja que no puede explicarse solamente con métodos epidemiológicos o análisis moleculares. Brindando servicios integrales a través del SSP, que incluyan pruebas de rutina, el enlace intensivo y la navegación del paciente, podría ser un modelo eficaz para lograr la supresión viral de las personas que se inyectan drogas.

## Introduction

Facilitated by the increase in opioid addiction and availability of synthetic opioids, injection drug use (IDU) associated HIV outbreaks have occurred across the globe [1]. Many opioids are injectable, promoting conditions that expedite the rapid transmission of communicable diseases, including HIV and hepatitis C (HCV), and exacerbating these serious public health epidemics as has been the case in the United States [1, 2]. Additionally, challenges in linkage to care, retention in care, and viral suppression in high risk people who inject drugs (PWID) are well documented, with estimates that less than half of PWID living with HIV in the US are virally suppressed [3, 4]. Further, as homelessness and participation in transactional sex are typically affiliated with PWID, disease transmission is compounded among such high-risk individuals [5, 6].

Evidence-based public health interventions can help mitigate disease transmission in these vulnerable populations. Yet, absence of interventions and delays in public health action have been connected with the incidence of IDU-associated HIV outbreaks [7]. Considerable evidence has shown that one of the most effective interventions for reducing HIV transmission among PWID is the provision of sterile syringes through syringe services programs (SSPs) [8, 9]. The use of medication-assisted treatment for substance use and dependence, rapid access to antiretroviral therapies (ART) for those living with a diagnosis of HIV, pre-exposure prophylaxis (PrEP), and other harm reduction methods are also effective at reducing HIV transmission [9], [10]. Greatest declines in the number of new HIV diagnoses following IDU-related HIV outbreaks were observed when SSPs were used in combination with other prevention interventions [1, 11].

Despite increases in rapid access to ART (“Test and Treat”) campaigns at the state and county level to reduce the incidence of HIV, Miami-Dade County, Florida, consistently has the highest rate of HIV diagnoses per 100,000 (42.9) in the US, with over 3.5 times the national rate (11.8) in 2017 [12]. In response to the prolonged HIV epidemic in South Florida, elevated healthcare expenditures associated with IDU, and concern about lack of proper venues for syringe disposal, the Florida legislature passed the 2016 Infectious Disease Elimination Act (IDEA), authorizing the state’s first SSP at the University of Miami [13], [14]. The program is limited to one-to-one syringe exchange by statute but also offers safe injection packs, nasal naloxone, harm reduction packs, HIV/HCV testing, referrals for healthcare, housing and drug treatment (including medication-assisted treatment), and linkage to HIV/HCV care and treatment.

Through December 20, 2018, the IDEA SSP has served 982 individuals, exchanged 253,096 syringes, distributed 1859 boxes of nasal naloxone, and administered 1576 screening tests for HIV and HCV. On February 19, 2018, the IDEA SSP implemented anonymous, opt-out, HIV/HCV rapid antibody screening of all participants at initial enrollment into the SSP, including ﻿subsequent testing for those testing non-reactive every 3 months thereafter. On March 9, 2018, the IDEA SSP identified its first anonymous rapid reactive HIV test in a participant who had previously tested non-reactive. Following two subsequent anonymous rapid reactive HIV tests among previously HIV non-reactive participants, the program notified the Florida Department of Health (DOH) on April 13, 2018. In total, ten anonymous rapid reactive HIV tests were identified at the SSP among previously non-reactive participants, suspected individuals with HIV seroconversion, over a 6-month period.

In response, the SSP partnered with DOH to provide on-site confidential testing and rapid linkage to HIV care and treatment. DOH initiated an epidemiologic investigation and embedded a Disease Intervention Specialist (DIS) within the SSP to facilitate contact tracing. DOH analyzed HIV-1 nucleotide sequence data to determine if the suspected individuals with HIV seroconversion were genetically similar based on the close relatedness of mutations arising along the genome of the virus at a genetic distance threshold of 0.5%, indicating recent and rapid transmission [15]. Molecular data can be used to understand transmission dynamics within a population as well as to provide opportunities for intervention [16]. Epidemiologically suggested HIV transmissions can be supported or uncorroborated using this molecular technique [17]. Previous investigations such as the Scott County, Indiana IDU outbreak in 2014–2015 used molecular HIV surveillance data reported to the health department to identify potential outbreak-related cases and subsequently established an SSP in response [18]. Further uses of molecular analysis of HIV-1 genotype sequences have been conducted, leading to an understanding of causal pathways and disease transmission [17, 19].

A multidisciplinary investigation between the IDEA SSP, local and state health departments, and the Centers for Disease Control and Prevention (CDC) was launched in order to (1) review and verify potential individuals with seroconversion for recent (in less than 6 months) HIV acquisition, (2) examine epidemiologic and molecular linkages of HIV diagnoses within a suspected HIV transmission network through confidential testing and contact tracing, and (3) assess care and treatment outcomes for identified individuals with HIV seroconversion.

## Methods

### Ethical Considerations

In accordance with both federal and state regulations for the protections of human subjects, the reporting of investigation findings was determined by the Florida Department of Health Institutional Review Board to be exempt from a full review.

### Case Definitions

A confirmed HIV seroconversion (case) was defined as an individual who had a documented negative HIV test followed within 6 months by a documented positive anonymous HIV test conducted at the SSP after February 19, 2018 through December 20, 2018, and who met the Council of State and Territorial Epidemiologists (CTSE) case definition for a reportable HIV case [20]. Furthermore, a primary epidemiologic link was defined as a sexual, syringe or works (injection equipment) sharing, or social connection with one of the cases over the past 12 months, identified through partner services interviews for locatable partners. Other individuals considered in the investigation were participants of the IDEA SSP known to be living with a previous diagnosis of HIV as of December 20, 2018 who had been reported to DOH.

### Epidemiologic Investigation

Anonymous HIV testing results at the IDEA SSP were recorded under an anonymous participant number, but reactive tests were not reportable to the state. Individuals with a suspected seroconversion identified by the SSP were verified following confidential testing to meet the CSTE case definition as a new HIV diagnosis [20]. Individuals with a confirmed seroconversion were assigned to a DIS embedded at the SSP for contact tracing and partner elicitation. Blood specimens for individuals with confirmed seroconversion were obtained, tested, and analyzed for molecular linkage. Epidemiologically-linked partners whose HIV status was negative or unknown were offered screening tests. For persons with a previous diagnosis of HIV, blood samples for those missing molecular sequences were collected. In the affected area, the SSP conducted daily outreach, increased syringe distribution, and collaborated with DOH to provide routine control measures such as rapid access to ART and screening of persons for HIV, syphilis, gonorrhea, chlamydia, and hepatitis B and C.

### Laboratory Testing

Individuals were screened for HIV infection using a point-of-care rapid HIV test (OraQuick ADVANCE^®^ Rapid HIV-1/2 Antibody Test, OraSure Technologies). Individuals with reactive rapid tests received confidential antibody screening (HIV-1/2 enzyme immunoassay) and confirmatory HIV tests (HIV-1/2 type-differentiating immunoassay). Other clinical labs including HIV-1 RNA/DNA NAAT (quantitative viral load), HIV-1 Genotype (protease/reverse transcriptase nucleotide sequence), and CD4 T-lymphocyte count were conducted. For persons who were virally suppressed (< 200 copies/mL), an HIV-1 resistance, proviral DNA (protease, reverse transcriptase, integrase inhibitors) test was performed.

### Patient Navigation and Rapid Access to HIV Care and Treatment

Patients with a reactive rapid HIV test were linked to care at the Miami-Dade County safety-net hospital, Jackson Memorial, via the DOH “Test and Treat” program to provide individuals with rapid access to ART and HIV care following initial diagnosis or when returning to care. Bictegravir-tenofovir alafenamide-emtricitabine (BIC/FTC/TAF) was the prescribed combination pill in the DOH “Test and Treat” program. The patients received a 30-day supply of BIC/FTC/TAF and had up to 60 days to complete financial eligibility and enrollment into the AIDS Drug Assistance Program (ADAP) to continue their prescribed therapies. To facilitate the linkage to care for this high-risk population, SSP staff engaged with case patients on the streets during outreach and accompanied them as they were seen by a Ryan White case manager, a clinician (physician or advanced practice provider), a phlebotomist for baseline laboratories, and a pharmacist for on-the-spot initiation of BIC/FTC/TAF.

### Measures

Individuals with confirmed seroconversion were compared to their epidemiologic linkages and SSP participants with a previous HIV diagnosis. Demographics (age, sex, race/ethnicity, and housing), HIV exposure risk, and syringe sharing status were obtained from DOH surveillance systems for HIV reporting and case management (enhanced HIV/AIDS Reporting System and Patient Reporting Investigation Surveillance Manager [PRISM]). Clinical characteristics and outcomes including baseline viral load and CD4 count, time to linkage to care, time to viral suppression, and coinfection or prior infection with syphilis and HCV were obtained for all linked individuals. Time to linkage to care was defined as the number of days between anonymous reactive rapid HIV test and first visit to an HIV clinic with a reported initial viral load. Time to viral suppression was defined as the total number of days between anonymous reactive rapid HIV test at the SSP and the specimen collection date for the first reported viral load of less than 200 copies/mL. In-care time to viral suppression was defined as the number of days between the date of first reported viral load and the first reported viral load of less than 200 copies/mL. HIV diagnosis date was defined as the earliest specimen collection date for a reported reactive HIV confirmatory test.

### Epidemiologic and Molecular HIV Network Analyses

Connections between individuals with HIV seroconversion and epidemiologic linkages as well as other SSP participants with a previous HIV diagnosis were visualized using the Harel-Koren Fast Multiscale algorithm in NodeXL Basic (Social Media Research Foundation) [21]. Molecular linkages, using the first reported nucleotide sequences after diagnosis within the reverse transcriptase and protease section of the Pol region of the HIV genome, were assessed at the 1.5% and 0.5% genetic distance thresholds using DOH local access to the Secure HIV Transmission Cluster Engine (TRACE) as previously outlined by Oster et al. [15]. Case nucleotide sequences were compared to 38,395 molecular sequences from Florida HIV diagnoses uploaded to Secure HIV TRACE. Importantly, although molecular HIV surveillance can identify HIV transmission networks experiencing recent and rapid HIV transmission, directionality (who transmitted the virus to whom) cannot be determined using molecular data.

## Results

From February 19, 2018 to December 20, 2018, seven individuals out of ten suspected patients with an HIV diagnosis met the case definition of an acute HIV seroconversion. The other three suspected cases had been previously diagnosed before implementation of the IDEA SSP’s universal testing protocol. Another 32 individuals participating in the SSP who were previously living with an HIV diagnosis were also reviewed for molecular linkages, seven of whom were also primary epidemiologic links to a confirmed case. An embedded DIS was able to interview 15 of these individuals, eight of whom DOH was previously unable to locate for investigation.

Confirmed cases had a median baseline viral load of 173,691 copies per mL (range 477–684,490) and median baseline CD4 count of 458 (range 305–885) (Table 1). None of the confirmed cases were HIV stage 3 (AIDS) at diagnosis. All seven individuals who seroconverted were homeless and injected heroin, often in combination with cocaine (speedball).

**Table 1:** Characteristics of individuals within the HIV transmission network and persons with diagnosed HIV participating in syringe services program, Miami, FL, 2018

	Individuals with HIV seroconversion (n = 7)	Primary epidemiologic links^a^ (n = 10)	IDEA participants living with HIV^a^ (n = 32)
Demographics			
Age—median(range)	39 (27–58)	33 (25–57)	39.5 (25–69)
Sex—n(%)			
Male	6 (85.7)	4 (40.0)	17 (53.1)
Female	1 (14.3)	6 (60.0)	15 (46.9)
Race/ethnicity—n(%)			
non-Hispanic White	5 (71.4)	6 (60.0)	13 (40.6)
non-Hispanic Black	0	1 (10.0)	8 (25.0)
Multi-race	1 (14.3)	0	1 (3.1)
Hispanic	1 (14.3)	1 (10.0)	10 (31.3)
Unknown	0	2 (20.0)	0
Homeless			
Yes	7 (100)	4 (40.0)	9 (28.1)
No	0	4 (40.0)	23 (71.9)
Unknown	0	2 (20.0)	0
Self-Reported HIV exposure risk^b^			
Heterosexual contact	1 (14.3)	3 (37.5)	7 (21.9)
IDU	5 (71.4)	4 (50.0)	14 (43.8)
MSM	0	1 (12.5)	4 (12.5)
MSM/IDU	1 (14.3)	0	7 (21.9)
Syringe sharing			
Yes	6 (85.7)	5 (50.0)	6 (18.8)
No	1 (14.3)	0	1 (3.1)
Unknown	0	5 (50.0)	25 (78.1)
Reported drug use			
Heroin	7 (100)	5 (50.0)	6 (18.8)
Cocaine	5 (71.4)	4 (40.0)	6 (18.8)
Methamphetamine	1 (14.3)	2 (20.0)	3 (9.4)
Crack	1 (14.3)	5 (50.0)	6 (18.8)
Speedball	2 (28.6)	2 (20.0)	2 (6.3)
HIV clinical characteristics			
Baseline viral load (copies/mL)—median(range)	173,691 (477–684,490)	37,473 (< 20–548,046)	13,207 (< 20–1,280,000)
Baseline CD4 count—median(range)	458 (305–885)	340 (143–914)	456 (8–1562)
Ever virally suppressed			
Yes	7 (100.0)	4 (50.0)	23 (71.9)
No	0	4 (50.0)	9 (28.1)
Currently virally suppressed^c^			
Yes	7 (100.0)	3 (37.5)	17 (53.1)
No	0	5 (62.5)	15 (46.9)
Time to linkage to care—mean days(range)	20 (0–49)	–	–
Time to viral suppression—mean(range)	70 (14–158)	–	–
Time to in-care viral suppression—mean days(range)	50 (14–125)	39 (0–96)	874 (0–6168)
HIV genotype—n(%)			
Yes	5 (71.4)	3 (37.5)	18 (56.3)
No	2 (28.6)	5 (62.5)	14 (43.7)
Hepatitis C antibody^d^—n(%)			
Positive before 2018	4 (57.1)	5 (62.5)	18 (56.3)
Positive in 2018	3 (42.9)	4 (50.0)	7 (21.9)
Unknown	3 (42.9)	1 (1.3)	11 (34.4)
Hepatitis C NAT^d^—n(%)			
Positive before 2018	1 (14.3)	3 (37.5)	8 (25.0)
Positive in 2018	2 (28.6)	3 (37.5)	5 (15.6)
Unknown	4 (57.1)	4 (50.0)	19 (59.4)
Syphilis^d^—n(%)			
Positive before 2018	2 (28.6)	3 (37.5)	12 (37.5)
Positive in 2018	1 (14.3)	2 (25.0)	5 (15.6)
Unknown	4 (57.1)	5 (62.5)	18 (56.3)

*IDU* injection drug use, *MSM* male sex with males

^a^Columns are not mutually exclusive, n = 7 individuals are represented in both groups

^b^Two individuals from the primary epidemiologic links category were excluded from the self-reported HIV exposure risk as they were not known to have HIV

^c^Virally suppressed as of December 20, 2018

^d^Rows are not mutually exclusive, individuals with reported labs could have had labs prior to 2018, in 2018 or both

### Patient Care Outcomes

**Fig. 1:**
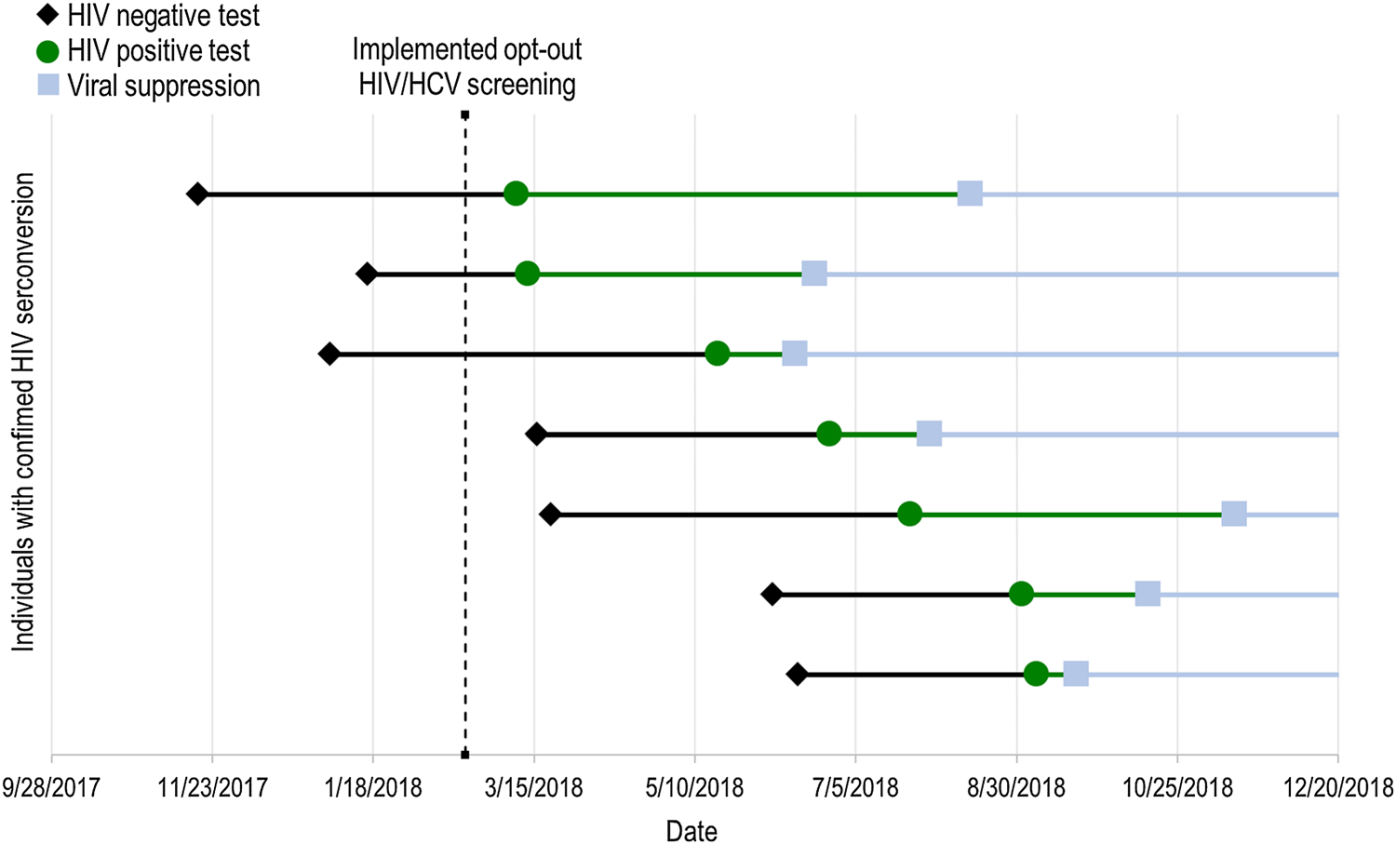
Case time-series of individuals with confirmed HIV seroconversion, Miami, FL, 2018. Black dots represent a documented HIV negative rapid antibody test at IDEA SSP. Green dots represent first documented HIV positive rapid antibody test at IDEA SSP. Blue dots represent documented viral load suppression (<  200 copies/mL)

All seven individuals with seroconversion were linked to care and achieved viral suppression with a mean time of 70 (14–158) days post anonymous HIV reactive result (Fig. 1). The mean time for patient linkage to care was 20 (0–49) days and time to in-care viral suppression was 50 (14–125) days. Only 53% (17 of 32) of the SSP participants with previously diagnosed HIV achieved viral suppression by the end of the investigation period.

### Transmission Network Dynamics

**Fig. 2:**
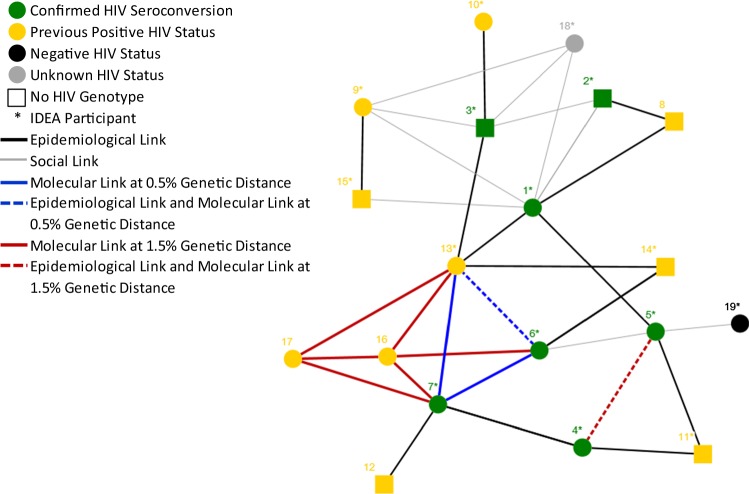
Primary epidemiologic and molecular network of individuals with confirmed HIV seroconversion, Miami, FL, 2018. Epidemiological links include sexual, needle or works (injection equipment) sharing connections between individuals in the primary network. Social links include social connections that do not include known sexual, needle or works sharing relationships between individuals in the primary network

Of the seven individuals with seroconversion, all are epidemiologically- and/or socially-linked (epi-linked) to at least two other individuals with seroconversion, with the exception of one individual who was only epi-linked to one other seroconversion (Fig. 2). Six of the seven individuals self-reported sharing syringes or works. Two individuals were epidemiologically-linked to individuals with previous HIV diagnoses through both syringe/works sharing and sexual contact. Two individuals with seroconversion and five individuals with primary epi-links self-reported exchanging sex for money or drugs. Resistance interpretations to HIV-1 antiretrovirals were obtained for all seven cases, but only five nucleotide sequences were able to be processed through Secure HIV TRACE for molecular linkage analysis.

Analysis of the five HIV genotypes revealed that two individuals with seroconversion (cases 6 and 7) were connected molecularly at 0.5% genetic distance, and another two individuals with seroconversion (cases 4 and 5) were molecularly linked at the 1.5% genetic distance (Fig. 1). Ten individuals were primarily epi-linked to the seven individuals with seroconversion, of which eight had previously diagnosed HIV (one negative and one of unknown HIV status). An additional nine risk partners of the seven were claimed but were not identifiable/investigated. In addition, eight of the ten epi-linked individuals were known IDEA SSP participants. Five of the eight previously diagnosed epi-linked individuals were missing genotype information (sequence or resistance interpretation), and of the remaining three, one (case 13) matched two individuals with seroconversion (cases 6 and 7) at the 0.5% genetic distance. Outside of the primary epi-linked network, two additional persons with diagnosed HIV (cases 16 and 17) matched two of the individuals with seroconversion (cases 6 and 7) and one epi-linked individual (case 13) at the 1.5% genetic distance, but had no reported epidemiological or social linkages to individuals in the risk network.

Resistance interpretation patterns and specific virus mutations for 13 of the 17 persons with diagnosed HIV (includes individuals with seroconversion) in the network were obtained (Table 2). Two individuals with seroconversion (case 6 and 7) and one primary epi-linkage (case 13) linked molecularly at the 0.5% genetic distance and shared 33 out of the 36 (92%) mutations identified, demonstrating high agreement between HIV-1 nucleotide sequences and HIV-1 resistance patterns. Moreover, two of the seven individuals with seroconversion (cases 2 and 3) did not have a reported genotype at the time of diagnosis and were virally suppressed at the time of the investigation. In an effort to obtain resistance patterns to aid in treatment, a proviral DNA test was performed and resistance mutations obtained. Neither of their resistance patterns had high agreement with any of the other 13 persons with diagnosed HIV.

**Table 2:** Genetic mutations of individuals with confirmed HIV seroconversion and associated primary epidemiologic and molecular linkages, Miami, FL, 2018

ID	Year of diagnosis	Year of genotype	Protease	Reverse transcriptase	Integrase
1	2018	2018	T12N, I15V, L19IV, L63P, I72V	E29D, V35T, K102N, S105A, K122E, D123N, S162Y, E169K, K173E, T200A, Q207E, R211K, E248EV, I257L, A272P, K277R, K281R, I293V	S17N, L45V, L101I, T124A, I141V, G163E, V165I, V201I, T218S, D278N
2	2018	2018	L63P	R211K, L214F	G163E
3	2018	2018	I13V, E35D, M36I, I62V, L63S, H69Q, A71T	R211K, L214F	None
4	2018	2018	E35D, N37D, K43R, L63P, I64L, I72IV, V77I, I93L	**K103N**, E6ED, V60I, R83K, D123E, I135RT, T200A, Q207E, V245K, K277R, I293V	NA
5	2018	2018	K20KR, E35D, N37D, D60E, L63P, I72V, V77I, I93L	**K103N**, V60I, R83K, D123E, K166R, T200A, V245K, K277R, A288S, I293V	E10D, S39C, L45Q, L101I, T122I, T124N, T125A, I203M
6	2018	2018	**M46L**, I13V, I15V, K20R, M36I, N37D, R41K, R57K, I93L	V35A, T39A, K122P, I135V, S162C, Q174K, D177E, T200A, Q207E, V276I, L283I, A288T, I293V	**E157Q**, S17N, V31I, I72V, E96D, L101I, T112A, T124A, T125A, K160Q, D167E, K215N, I220L, S283G
7	2018	2018	I13V, I15V, K20R, M36I, N37D, R41K, R57K, I93L	V35A, T39A, V118I, K122P, I135V, S162C, Q174K, D177E, T200A, Q207E, L283I, A288T, I293V	**E157Q**, S17N, V31I, I72V, E96D, L101I, T112A, T124A, T125A, K160Q, D167E, K215N, I220L, S283G
9	2016	2018	T12S, K14R, L19Q, R41K, I62V, L63P, Q92K	V35I, K122E, D123E, I135K, S162A, I195L, T200A, I202V, Q207E, A272P, K277R, V292I, E297A	K14R, A21S, V32I, A38S, S39C, M50I, I72V, L101I, T124A, K136N, V201I, A205S, T206S
10	2016	2017	I13V, E35D, M36I, R41RK, K45R, R57RK, I62V, L63HPSY, H69Q, A71T, I72V	V35M, V60I, R83K, K122KE, G196E, I202V, R211K, V245E, E248ED, A272P, K275KR, K277R, Q278H, E297Q	NA
13	2017	2018	I13V, I15V, K20R, M36I, N37D, R41K, R57K, I93L	V35A, T39A, V118I, K122P, I135V, S162C, Q174K, D177E, T200A, Q207E, L283I, A288T, I293V	**E157Q**, S17N, V31I, I72V, E96D, L101I, T112A, T124A, T125A, K160Q, D167E, K215N, I220L, S283G
14^a^	2017	2017	G16E, I62V, L63T, H69Y, V77I	NA	S17N, D41E, I72V, A76G, L101I, T112M, I113L, T124A, T125A, K156N, H171Q, V201I, A205S, T218S, I220L, Q221H, D278N, S283G
16	2016	2016	I13V, I15V, M36I, R41K, R57K	V35A, T39A, K122P, I135V, S162C, Q174K, D177E, T200A, Q207E, V245VE, L283I, A288T, I293V, E297EK, K311R	NA
17	2011	2011	I15V, L19LV, M36I, R41K	V35T, K122P, I135V, S162C, D177E, T200A, Q207E, L283I, I293V	NA

Bolded mutations represent major mutations associated with drug resistance to classifications of antiretroviral treatments

^a^Resistance patterns were reported for this individual, however a molecular sequence was unavailable for comparison in secure HIV TRACE

## Discussion

Early identification of individuals with HIV seroconversion among PWID in Miami, most of whom use both opioids and stimulants known to increase injection frequency and high risk behaviors [22], helped drive collaboration between a privately funded SSP and the health department at the county and state levels to investigate the HIV transmission network in Miami. With the HIV outbreak and response in Scott County, Indiana, investigators determined that early surveillance efforts could reduce emerging epidemics [18, 23]. In Miami-Dade County, the implementation of routine screening at the SSP, early identification of individuals with recent seroconversion, and subsequent investigation helped establish low-threshold routes of access to HIV care for PWID to mitigate further transmission of HIV within the community.

Our investigation yielded a complex HIV transmission network that included multiple modes of HIV exposure (sexual and syringe/works sharing) and transmission, both within and outside the SSP network, that could not be defined through either traditional epidemiological practices or molecular analyses alone as each source was incomplete in defining relationships within the network. This is in contrast to the Scott County, Indiana investigation where nearly 99% of the cases were highly-related by phylogenetic analysis but similar to a national level molecular evaluation where only 34% of high-risk partners were determined to be potential transmission partners [18, 24]. The proportion of individuals in the identified network reporting both sexual and IDU HIV exposure risk is one explanation for the lack of molecularly-linked cases through the SSP. The attributable fraction each of these exposure methods represents is unknown in this network but previous studies suggest that sexual risk in this cohort could have an increased impact on transmission and acquisition of HIV [25, 26].

The complex HIV transmission dynamics also highlighted the important role both traditional partner contact tracing and molecular data play to supplement information and build these epidemiological networks. Previous studies are in agreement that complementing traditional contact tracing with molecular HIV sequence data can confirm or refine transmission networks, and improve precision of HIV prevention activities [15, 24]. Furthermore, we were able to use HIV-1 resistance patterns to supplement for two missing nucleotide sequences among individuals with recent seroconversion since we observed high agreement in the number of mutations present for individuals with seroconversion linked at 0.5% genetic distance. Pooled studies observe the mutation accrual rate for HIV to be between 0.014 and 0.039 mutations per month or 2% per year [27], [28]. Therefore, we can suggest the use of resistance patterns to support the building of recent and rapid transmission networks for acute HIV infection.

In Miami, response efforts were coordinated in collaboration between the SSP and the Department which led to the organized response being delivered through the SSP. Early diagnosis and immediate ART initiation was prioritized to rapidly decrease viral transmission, reduce the impact of a potential outbreak, and improve quality of life [29–31]. Established “Test and Treat” paradigms have shown decreased time to virologic suppression, the ultimate goal in controlling further HIV transmission among PWID in Miami [32, 33]. The high linkage to care and viral suppression rates among the seven individuals with seroconversion were much greater compared to previous outbreaks of HIV among PWID. For example, during an HIV outbreak among PWID in Greece, linkage to care and initiation of ART through a “Test and ‘Treat”-like program was only 48.4% and 24.5%, respectively [11]. The second-generation integrase-based regimen was well tolerated by PWID and allowed SSP patients to achieve rapid in-care viral suppression. However, PWID with previously diagnosed HIV may require a more intensive intervention to achieve similar outcomes as PWID with recent HIV seroconversions.

Medication management at the SSP likely also contributed to rapid viral suppression in patients with acute HIV. SSP staff obtained third party authorization to pick up the patients’ single tablet regimen and provide pill boxes and safe storage of medications at the SSP. Furthermore, SSP staff conducted outreach, monitored appointments, and provided transport to healthcare services. This intensive linkage and active follow-up also included counseling by licensed mental health counselors at the SSP who administered evidence-based interventions aimed at increasing the patients’ interpersonal skills and self-efficacy [34]. Patients also had the opportunity for consultation with an HIV physician available on-site weekly. Onsite services at the SSP likely played a role in increasing trust in the medical staff. Miller et al. recently reported that intensive and flexible patient navigation with psychosocial counseling was central to increased initiation of ART and viral suppression as well as decreased transmission among PWID [35]. The flexibility and continuous process improvement during the investigation led to design of a culturally appropriate navigation program for a challenging patient population in a complex healthcare system.

Increased availability of syringes through outreach trips to the geographic area of the transmission network was also prioritized to decrease HIV transmission among homeless PWID. These outreach trips were enhanced through partnership with the local health department for publicly funded services for additional screening and testing and adaptation of the “Test and Treat” program for the population. This coordination of care allowed the SSP and DOH to overcome recognized barriers to HIV care and retention in an at-risk population within the Miami community [36].

Similarly to the Scott County, Indiana outbreak, a large proportion of the HIV seroconversions were also co-infected with HCV. In Florida, if eligible, the AIDS Drug Assistance Program (ADAP) will follow the American Association for the Study of Liver Diseases (AASLD) Guidelines for HCV treatment for those co-infected with HIV. There are no current requirements related to sobriety or drug use other than the clinician must feel the patient can be compliant with treatment. However, for this study the treatment and medical records pertaining to the treatment of HCV were determined outside of the scope of the investigation.

One limitation was the inability to obtain genotypes for two of the seven individuals with HIV seroconversion and five of the eight epi-linked partners, which might have led to missed potential linkages within the SSP network and had to be supplemented with HIV-1 resistance patterns. However, the scarcity of molecular linkages between the individuals with seroconversion and persons with diagnosed HIV and a reported genotype using the SSP, despite epidemiologic connections, suggests that these seroconversions among PWID are likely transmissions occurring outside the SSP network. Secondly, due to the small number of HIV cases, the outcomes of our patient navigation model need to be validated among a larger cohort over a longer timeframe. However, the goal of this investigation was to limit the number of new HIV transmissions and mitigate ongoing HIV transmission by rapidly applying and adapting interventions during the investigation. Lastly, the epi-linkages are all based on self-report and could be subject to social desirability bias. Embedding the DIS within the SSP likely facilitated patient ease and mitigated bias during the partner services interviews.

Despite these limitations, to our knowledge this is the first study to show such rapid viral suppression in PWID by introducing an expanded and integrated model of care delivery based at an SSP. The SSP played a central role in testing and surveillance due to the trusted relationship with the PWID population. Unfortunately, previously diagnosed cases among persons living with HIV did not have equivalent clinical outcomes, suggesting the importance of early engagement at an SSP, linkage, and navigation in this population. Implementation of universal HIV/HCV testing at the SSP provided a high yield venue to aid in the rapid identification of HIV seroconversions among PWID. The SSP thus played a formative role in response to a number of recent HIV seroconversions in Miami. Other jurisdictions experiencing HIV seroconversions could benefit from partnerships between their local SSPs, health departments and medical systems in order to bring timely and appropriate linkage to medical care and disease intervention. In Miami, the early diagnosis, linkage to care, and viral suppression potentially averted new HIV transmissions in this network as well as within the surrounding community given the complex network dynamics involved.

## Conclusions

Overall, this SSP’s role in the collaborative effort led to early detection and suppression of a typically medically underserved and marginalized population, limiting the transmission of HIV to other individuals. Providing concentrated wrap-around services at an SSP including implementing routine screening and offering intensive linkage and patient navigation could be an effective model for identifying new transmissions, engaging individuals into care, achieving viral suppression in PWID, and working towards overall community harm reduction. Ultimately, to prevent HIV seroconversions, proven upstream interventions like clean syringe access and wrap-around PrEP access need to be implemented or expanded within these communities.
